# Exploring temporal patterning of psychological skills usage during the week leading up to competition: Lessons for developing intervention programmes

**DOI:** 10.1371/journal.pone.0181814

**Published:** 2017-08-07

**Authors:** John Elvis Hagan, Dietmar Pollmann, Thomas Schack

**Affiliations:** 1 "Neurocognition and Action - Biomechanics"- Research Group, Faculty of Psychology and Sport Sciences, Bielefeld University, Bielefeld, Germany; 2 Center of Excellence "Cognitive Interaction Technology" CITEC, Bielefeld, Germany; Sao Paulo State University, BRAZIL

## Abstract

**Background and purpose:**

Although sport psychology literature focuses on psychological skills use to promote proficiency, it is still puzzling that current research has focused on psychological skills use only during competition. There remains a scarcity of empirical evidence to support the timing, and content of psychological skill application during the time preceding competition. This study examined the extent to which psychological skills usage are dynamic or stable over a 7-day pre-competitive period and whether any natural learning experiences might have accounted for the acquisition of these skills across gender and skill level.

**Methods and results:**

Ninety elite and sub-elite table tennis players completed the Test of Performance Strategies (TOPS) at three different periods (7 days, 2 days, 1 hour) before competition. A MANOVA repeated measures with follow-up analyses revealed significant multivariate main effects for only skill level and time-to-competition with no interactions. Specifically, elite (international) athletes reported more usage than sub-elite (national) counterparts for self-talk, imagery and relaxation respectively. Time-to-competition effects showed imagery use decreased steadily across the three time points while reported usage of relaxation were almost at the same level on two time points (7 days and 1 hour) but decreased 2 days before competition.

**Conclusions:**

Findings suggest an implementation of formalized and periodized psychological skills training programs over continuous training cycles. This may foster a positive long-term athletes’ psychological state prior to the onset of competition.

## Introduction

One sport that triggers immense emotional coloration on performers by virtue of its task complexity and competition related demands is table tennis. Like other quick interactive sports, performers in table tennis are required to use advanced signals or cues from their immediate playing environment to determine what movement actions ought to be calibrated and displayed in reaction to often high speed balls played within a limited time window. For instance, the actual play time between two opposing players in a game of table tennis after all interruptions deducted could last between 3 to 5 minutes [1–2]. Hence, any negative emotional episode may psychologically destabilize the player and cause faulty movement actions, affecting quality of play and subsequent game outcome. Therefore, players’ mastery of game situations depends highly on the quality of psychological techniques utilized than on athletic abilities [1–2]. Consequently, players’ ability to combat these emotional experiences using different psychological skills might determine more than half of their chances to win especially when competing against opponents of the same level (skill, experience; [3]). For example, some research have shown that elite athletes deploy a range of basic psychological skills in comparison to their sub-elite counterparts in attempt to improve their psychological states toward performance related tasks [4,5]. Additionally, males and females may also experience different types and level of stressors that may require different psychological skills for successful resolution. Anshel and associates acknowledged that sex differentiates the selection of coping strategies in the general psychology literature, yet it has received scant attention from researchers in relation to competitive athletes [6]. Therefore, if participating in competitive sport environment often places these athletes under undue intense physical and psychological demands in the form of anxiety and other emotional experiences, then these arduous challenges require athletes not only to use automated technical and tactical skills but also to develop and employ an arsenal of psychological (cognitive and behavioral) skills to achieve performance success and satisfaction [7–9].

The importance of psychological skills has triggered considerable research, especially in elite sports [10]. From applied standpoint, there is the need for practitioners such as sport psychology consultants, coaches, and performers to emphasize effective psychological preparation during the time preceding competitive events [11]. Effective preparation for sport performance is crucial in helping the individual cope with the pressures and stresses in any performance context. The maintenance of an optimal psychological state during the pre-performance period in particular, has been highlighted as a key determinant on performance [12]. Although sport psychology literature focuses on psychological skills use to promote proficiency, it is still puzzling that research has primarily focused on psychological skills use during competition [13]. However, there remains a dearth of empirical evidence to support the structure, timing, and content of psychological skill application during the time preceding competition; a severe limitation for a theory-to practice [11,14–15].

Four basic psychological skills commonly investigated in sport psychology research are goal setting, self-talk, imagery, and relaxation skills [16]. Goal-setting has consistently featured in applied sport psychology literature and mostly proven that divergent goals (e.g., outcome, performance, and process) can impact sport performance by triggering variations in athletes’ levels of attentional focus, self-confidence, effort, and motivation [17]. Also mentioned is relaxation that includes both unstructured [16] as well as more structured methods that are categorized into two groups: muscle-to-mind (e.g., progressive muscular relaxation) and mind-to-muscle (e.g., transcendental mediation). This basic psychological skill is seen to be crucial in controlling activation and arousal levels [18]. Another psychological skill that has recently gained much prominence due to its conceptualization and multiple functions is self-talk [19–20]. Often seen as “a multidimensional phenomenon concerned with athletes’ verbalizations that are addressed to themselves” [20], p. 905. These verbalizations have been shown to have both cognitive and motivational functions [19]. Imagery has also become a popular technique that has received a considerable research attention [21–22]. Imagery affects athletes’ schematic structure of selected movements as well as their psychological states in terms of both physiological and psychological adaptations [23]. Specifically, it enhances athletes’ aspects of performance, including refining skills and strategies, regulating emotions, and activation levels, and managing cognitions, and motivational drive [24–25].

Hardy, Jones, and Gould [16] asserted that psychological skills can be applied in a single (i.e., basic psychological skills such as imagery or self-talk) or combined fashion (i.e., advanced psychological [multi-modal)] skills such as pre-competition routines that often integrate imagery and/ or self-talk). Even though researchers have identified advanced psychological strategies to have diverse significant impacts on emotional experiences [15,26–27], care must be taken in order not to undermine the importance of assessing the use of basic psychological skills. According to Wadey and Hanton [28], examining their collective impact would bring setbacks when assessing their separate effects. Aside these illustrations above, there is recognition that these psychological skills on expert performance in sport are acquired and developed either through educational intervention experiences via psychological skills training programs [15,28–29], and/ or natural learning experiences [26,30–31].

Psychological skills usage at practice, not just in competition, is very crucial. For example, Frey, Laguna, and Ravizza [29] showed that athletes’ use of psychological skills was associated to their seeming accomplishments during practice and in competition. According to these authors, the more use of these psychological skills by athletes prior to competition, the more likely they would perceive themselves as successful, not only prior to competitive engagements, but also during competition. Frey and associates admonished sport psychology practitioners to create awareness on the relationship between psychological skills use during practice and success in competition for sport coaches. Athletes would be encouraged to use them when practicing their physical skills in an attempt to enhance their quality of practice and subsequent performance. Most coaches recognize the value of psychological skills in competitive sporting environment, yet these are often devalued during technical preparations prior to competition [32].

Taken together, there seems to be a paucity of research regarding the extent to which skill level and sex interact as a function of psychological skills using the time-to-event paradigm. To date, little research evidence exist on the use of pre-event psychological skills to deal with emotional episodes that unfold over time especially when the experience of the individual performer is often expected to evaluate critical situations and calibrate the required responses [8,14,33]. Of the studies that exist, the number of truly elite athletes across sex has always been limited and their applicability to real performance environments have been questioned [34–35]. Few studies have shown that sex differentiates the selection of psychological skills in relation to competitive athletes [36–37]. Given that psychological skills usage have demonstrated positive impacts on athletes in numerous sport such as swimming [38], soccer [39], Hockey [15], softball [40], skating [41], golf [42], and tennis [43], an empirical enquiry into table tennis in a natural field (ecologically valid) setting could also provide further research information on the timing of psychological skills designed to assist different standard of performers affected by their pre-event psychological states.

The purpose of the present study was to establish the extent to which skill level and sex interact across psychological skills over a 7-day pre competitive period. Given that research has been able to distinguish elite and sub-elite athletes based on their use of psychological skills, it will be interesting to ascertain whether this will hold across another affective variable (male and females). It was hypothesized that elite athletes would show greater (more) usage of psychological skills, including goal setting, imagery, and self-talk [5,26,31,44], while sub-elite performers would report greater relaxation skill usage [4] across sex and temporal period. In addition, due to fact that the study population has no formal experience with psychological skills training, it was proposed that any reported psychological skills across skill and sex might have been acquired and developed through natural learning experiences. It is also anticipated that the differences in psychological skills usage across skill level and sex would remain stable throughout the pre-competition period.

## Materials and methods

### Participants selection criteria

The purposive sampling technique was used for the selection of study participants who met the criteria toward this research direction [45]. A two-step procedure was implemented to meet this requirement. For elite (international) status, participants should have attained national recognition and competed for Ghana in some international competitions in the course of their playing career. The criteria for sub-elite (national) status was based on participants who had attained regional and university/college awards and have continuously played in the national table tennis league for over four consecutive seasons at the time of the data collection, an approach used in a similar study [46].

#### Participants

The Institutional Review Board (IRB) of Bielefeld University approved this study through the adherence of the ethical standards of the sixth revision of the Declaration of Helsinki. Establishing links with the performers who competed in the national league were done through their respective sport clubs after contacting the Ghana National Sports Authority and National Table Tennis Association. Performers who met the elite criteria were communicated to and subsequently informed about the rationale of the study, which was to gain a deeper insight into how they respond to their emotional responses with the application of psychological skills during their preparatory period. Selected performers gave their written consent after being given assurance that their confidentiality and anonymity were going to be preserved at all stages of the data collection process and that data collected were solely for academic reasons. Performers were also informed that their participation was entirely voluntary so they could discontinue responding to the survey instruments at any moment they felt like doing so. The sample size was ninety (N = 90), with players’ ages ranged from 15 to 39 years (*M* = 26.26, *SD* = 5.29). Athletes, N = 35 (38.9%) were females while N = 55 (61.1%) were males. Further, athletes’ classification, N = 47 (52.2%) was revealed as elite (international), with N = 43 (47.8%) noted as sub-elite (national), all with at least 3 years of competitive experience (*M* = 9.63, *SD* = 5.12), train on the average, four times a week.

### Instrumentation

#### Test of performance strategies (TOPS)

The TOPS [13] evaluates 16 psychological skills used by athletes during both practice (training) and in competition. The idea behind the use of TOPS was because none of the previously reviewed instruments has measured psychological skills use in both practice and competition and has four items on each subscale. Space precludes a considerable review of the TOPS inventory, cf. [13]. For the purposes of the current investigation, the practice subscale was assessed on relaxation, goal-setting, imagery and self-talk skills prior to competition. As a result, other subscales items were not included in the present study. An approach adopted in similar studies, e.g., [4–5]. Examples of items prior to competition included for relaxation “I practice a way to relax” and for goal-setting “I set realistic but challenging goals for practice”. Items for imagery had “I rehearse my performance in my mind before practice”. Self-talk included “I talk positively to myself to get the most out of practices”. The frequency of each item on a scale was ranked by participants from 1 (“never”) to 5 (“always”), with overall psychological skill scores ranging from 4 to 20 by summation. The initial reported Cronbach alpha coefficients are 0.80 for relaxation, 0.78 for goal-setting, 0.79 for imagery and 0.80 for self-talk [13]. Encouraging construct validity has been reported [47]. A criterion score of 15 was chosen to represent high usage for all the four scales based on previous research on TOPS [4–5,28]. Cronbach alpha reliability analysis was performed on the present data, with reported alpha coefficients as follows: .81, self-talk; .81, goal setting; .80, imagery; .80, relaxation. Cronbach coefficients values of .70 or higher are generally deemed acceptable for reliability analysis.

### Procedure

TOPS was measured at three different stages (7days, 2 days, 1 hour) in a 7-day interval prior to a competitive fixture. To avoid any contextual influences (e.g., audience effect), an introductory session was held to brief participants on the concept of psychological skills. Educating athletes about emotional responses can help recognize, distinguish, and accurately report on their experiences [48–49]. This briefing is similar to other previous temporal based research examining anxiety responses during a 7-day competitive cycle [15,50]. Participants were required to note their psychological skills implemented in an attempt to cope with perceived negative emotional experiences they might encounter during the pre-competition period. Prior to completion, participants were presented with standardized instructions based on the recommendations of Smith et al. [51] and Thomas et al. [13] respectively. These authors emphasized the need for confidentiality of responses and to consider each item on its own merit, thus attempting to minimize social desirability, accentuate honesty, and emphasizing that there was no right or wrong answers.

### Data analysis

The data analysis was divided into different stages. Data screening procedures were first done to determine the data accuracy and statistical assumptions (univariate and multivariate analyses, including missing cases). A follow-up descriptive statistics and Pearson correlations analyses were conducted to show the association between the subscales. Next, the potential impact of skill level and sex on TOPS subscales using Multivariate Analyses of Variance (MANOVA) repeated measures were computed, testing for interaction and main effects. Specifically, a 2 (skill level) × 2 (sex) × 3 (time-to-event) MANOVAs were done. One MANOVA was conducted on four psychological skills, with skill level and sex acting as the independent variables while self-talk, goal-setting, imagery and relaxation acted as the dependent variables over all time periods in the analysis. Additionally, follow-up univariate analyses of variance (ANOVA) with Bonferroni adjustments for TOPS subscales were employed to determine where potential differences could be identified [52–53]. A Mauchly’s test for the within-subject repeated measure analysis on the sphericity assumption was also computed, and whenever the test was violated, a Greenhouse-Geisser test was done for the necessary technical corrections [52–53].

## Results

### Data pre-screening

Data were tested for missing cases, distributions, and assumptions of univariate and multivariate analyses. However, no missing cases and univariate or multivariate outliers were revealed through Mahalanobis distance test. Similarly, normality, linearity, multicollinearity, and singularity assumptions were deemed appropriate. Again, the equality of covariance matrices assumption, even though satisfactory at the univariate level (Levene’s test and F_max_ ratios), was violated in some cases at the multivariate level (Box’s test). Hence, the appropriate multivariate test statistic chosen for reporting due to its robustness over violations was Pillai’s trace [52–53].

#### Correlational analysis for selected TOPS practice subscales

Correlations among each of the TOPS practice subscales are illustrated in Table 1. Moderate pattern of relationships occurred between all the psychological skills. The most prominent correlations emerged between self-talk and imagery (r = .51, p < .01), relaxation and self-talk (r = .49, p < .01), goal-setting and self-talk (r = .48, p < .01) and imagery and goal-setting (r = 45, p < .05). Relaxation and imagery recorded the lowest correlation (r = .32, p < .01).

**Table 1:** TOPS practice subscale correlations.

Variable	ST	GS	IM	RE
Self-Talk (ST)				
Goal-Setting (GS)	.48**			
Imagery (IM)	.51**	.45*		
Relaxation (RE)	.49**	.34	.32**	

**. Correlation is significant at the 0.01 level (2-tailed).

*. Correlation is significant at the 0.05 level (2-tailed).

#### Impact of skill level and sex on psychological skills scores

Across all analyses no interaction effects were noted (p > .05), suggesting that any change-over-time patterns were similar across gender and skill level. However, the results revealed significant main effects for between-subjects on the combined psychological skills across skill level were noted, Pillai’s trace = .198, F (4, 83) = 5.127, P < .05, partial eta squared = .99 and not for sex, Pillai’s trace = .012, F (4, 83) = .243, P > .05, with a follow-up ANOVA noting significance for self-talk, F (1, 86) = 18.974, p < .05, partial eta squared = .88; imagery, F (1, 86) = 7.232, P < .05, partial eta squared = .67 and relaxation, F (1, 86) = 5.098, p < .05, partial eta squared = .55. As a result, data was collapsed into skill level classification for the changeover-time analysis. An inspection of the mean scores reveal that elite (international) athletes reported more usage than sub-elite (national) counterparts for self-talk (M = 16.06 vs 13.97), imagery (M = 13.95 vs 12.70) and relaxation, (M = 10.99 vs 9.74) respectively throughout the preparation phase as competition approached.

#### Descriptive statistics

**Fig 1:**
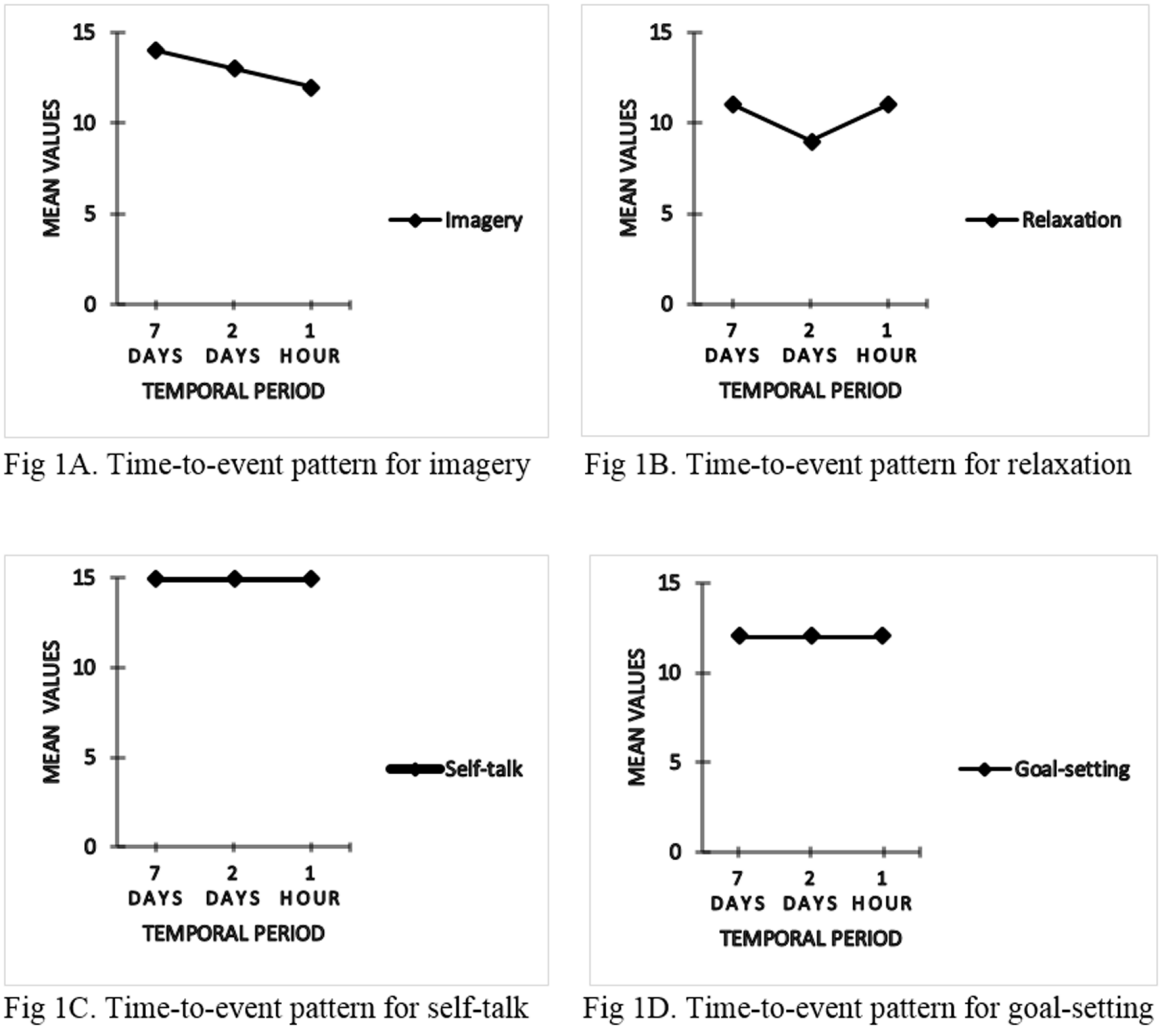
Time-to-event patterns for psychological skills (imagery, relaxation, self-talk, and goal-setting) collapsed across skill level.

Time-to-event main effects were also noted, Pillai’s trace = .513, F (8, 79) = 10.398, p < .05, partial eta squared = .51. A follow-up within-subjects ANOVA showed changes for imagery, F (2, 86) = 19.106, p < .05, partial eta squared = .88 and relaxation, F (2, 86) = 5.780, p < .05, partial eta squared = .66. A corrected t tests show that reported usage of imagery decreased steadily across the three time points (7 days, 2 days, 1 hr) preceding competition while reported usage of relaxation were almost at the same level on two time points (7 days and 1 hr) but decreased 2 days before competition (see Table 2; Fig 1).

**Table 2:** Means and standard deviations for TOPS practice subscale scores collapsed across skill level for all time periods.

Variable	7 days M (SD)	2 days M (SD)	1 hr M (SD)
Self-talk	15.27 (2.87)	15.09 (2.75)	14.91(2.87)
Goal-setting	12.21 (1.99)	12.78(2,14)	12.43 (1.89)
Imagery	14.09 (2.22)	13.17(2.57)	13.37 (2.44)
Relaxation	10.40 (3.22)	9.87(2.91)	11.01 (3.24)

## Discussion

The central focus of the present investigation was to explore the potential differences that exist in the psychological skills usage among elite sample of performers across sex during a one week preparatory phase as competition approached and determine whether reported usage of these strategies are stable or dynamic prior to a competitive fixture. Across all analyses no interaction effects were noted (p > .05) suggesting that any change-over-time patterns were similar across skill level and sex. However, significant main effects were noted for only skill level and time-to-event, partially supporting the hypotheses formulated.

Results from the correlational analysis revealed that the four practice subscales confirm that TOPS is an internally stable instrument, with a relative degree of independence between the different psychological constructs. The moderate correlations among the psychological skills suggest that athletes tend to use one or more of the practice strategies to potentially impact on their psychological states as competition neared. Furthermore, findings also suggest not all of these skills were used by all participants, reinforcing the notion of individual differences in the selection of psychological skills [54]. Elite (international) performers deployed self-talk, imagery, and relaxation skills in an attempt to maintain a positive psychological states prior to upcoming performance [2]. Research has clearly shown that elite competitors differ from their sub-elite counterparts on a number of psychological skills and attributes [16]. Elite athletes in this study scored highest on self-talk but recorded moderate mean values for imagery and relaxation compared with the sub-elite (national) counterparts. The reported mean values for imagery and relaxation skills did not meet the criterion score of 15 proposed by some researchers [4–5,28]. Higher self-talk score noted with the current sample was not surprising in that, table tennis like most rackets games, it is more prevalent to see elite performers use single cue words, phrases, or full sentences, and specific or general task instructions to more or less superimpose their dominance over their opponents. Also, given the elite status of athletes, it is surprising that elite performers reported moderate overall mean scores for imagery and relaxation compared to those found by Thomas and associates [13]. Although the current study was not designed to follow an intervention approach, it is assumed that the reported use of the aforementioned psychological skills could positively favor cognitive processes such as planning, reasoning, analyzing their situations, while anticipating future actions [55] prior to a competitive fixture. As self-regulation skills, we believe reported usage would help elite athletes’ manage thoughts, feelings, and behaviors by being proactive in recognizing and maximizing opportunities inherent in their training as competition approached. These findings indicate that both elite and sub-elite table tennis performers used a wide range of similar strategies, even if variations between the two groups of athletes exist. The elites however displayed a high ability on these strategies, on the assumption that they are likely to improve their self-regulation than sub-elite counterparts [56].

The hypothesis regarding the medium of psychological skills acquisition was confirmed. From an applied perspective, sport psychology consulting is at its nascent stage, development, and recognition in Ghana. Currently, applied work with elite athletes is mainly preserved for football, which for almost a decade, have engaged the services of a professional sport psychologist. This means that in table tennis, like other less endowed sport, elite performers appear to develop their psychological skills over the years through natural learning experiences and social influences without any involvement in any formalized structured psychological skills training program. This process might have unfolded throughout these athletes’ playing career through perhaps diverse mechanisms like heeding to parents, friends, coaches, and more experienced competitors’ advice, and competing at different levels of competition and against different competitors with varied skills [26,30]. The significance of social background (i.e., family, coach, and exposure to elite athlete models) in the long term athlete development plan (LTAD; talent identification) has previously been emphasized [31]. Gould [57] reiterated that when an athlete is exposed at an early age to elite performers in the same sporting discipline was one factor among others which “provided both inspiration and various forms of vicarious learning” [p. 58]. These scenarios might have accounted for the psychological skills reported by the current sample of athletes which is even mirrored by moderate mean values recorded.

In partial support to the proposed hypotheses, time-to-competition changes were observed on some psychological skills (imagery and relaxation, see Fig 1A and 1B), supporting the dynamic nature of a coping process. Despite performers reported usage of imagery skills, this steadily decreased across the temporal period prior to competition. If imagery skills are fundamental to refinement of routines that are usually used to replace natural occurring negative images experienced due to reflecting back on mistakes made in performance while in practice about some technical skills, then the reported decrease over time should be of concern. There is a possibility that elite athletes’ ability to restructure or reconstruct a positive experience as corrective mechanism of a hitherto wrongly executed skill due to perhaps poor technical awareness may have been compromised [15]. These explanations further suggest that the schematic structure of selected course of actions or movements that stabilize the motor representation structure (motor program) in the long-term memory and the priming of neural pathways that improve movement or performance outcomes are likely to be negatively affected [23,58]

Additionally, throughout the 7-day temporal period, athletes (elite/sub-elite) spent time per week on relaxation skills. A plausible explanation is that elite athletes usually experience more intense pressure to perform because they compete at higher level with greater competitive demands. These athletes may perceive relaxation as more relevant to competing effectively, hence spent more time using relaxation to calm their nerves [59–60]. Also, the brevity of response window in table tennis which often dictates opponent’s reactive play requires advance cues and anticipatory movements in a relaxed manner. Therefore, small amount of pressure from the environment may negatively affect the neuromuscular control mechanisms and subsequently on performance, hence the use of relaxation technique. Again, performance in discontinuous tasks of short-duration and intervals between trials like table tennis, is more influenced by physiological reactions (tense muscles) than continuous tasks like soccer, rugby and other team events that allow somatic reactions to return to stable levels as players become more involved in the activity (i.e., as game progresses; [61]). Therefore, relaxation skills were used to possibly promote recovery between interval phases within practice and that these skills are likely to be repeated while in competition. Researchers have proposed that relaxation can be used as a means of psychological and physical recovery following practice or competition [59,62]. Proven already in clinical psychology is the fact that relaxation might aid recovery via a physical pathway, as interventions have been shown to speed healing and reduce the negative effects of stress on the immune system [63].

We also argue that although both imagery and relaxation skills can be acquired intuitively due to their inherent characteristics as shown in this study, the inability of these athletes to maintain their usage across the temporal period and the values reported questions how elaborate and effective these skills were developed over time. This situation could potentially impede or harm future performance. The non-significant main effects noted for self-talk and goal-setting suggest stability of these psychological skills during the temporal phase (see Table 2; Fig 1C and 1D). The intermittent breaks between trials could have also triggered frequent verbalizations or cue words as self-talk. These words are self-generated in a natural manner to deal with both movement and stroke pattern related problems, commonly associated with racket sport like table tennis [64–65]. This finding is mirrored by the high mean values obtained and maintained across the preparatory phase. Another assumption is that goals impact on performance outcomes by centering attention on task-relevant cues especially when athletes are trying to meet challenging objectives towards an important event [66]. This centering may potentially help athletes to continuously and strategically plan for their future event, as such might have played on their minds throughout preparation period. However, the low means values challenges their effectiveness.

Although the findings are somewhat encouraging, empirical studies have also shown that formalized psychological or mental skills training programs are very effective in fostering greater and more elaborate psychological skills in athletes from a range of sports [15,38]. These carefully planned interventions render athletes autonomous by enhancing their effectiveness and functioning with or without the support of the coach, psychologist or any other analogous personnel. There is the need to integrate psychological skills training programs that should typically include a combination of programs with specific individual needs of athletes in table tennis and perhaps other sport disciplines in Ghana. These programs should be periodized over consecutive training cycles where education will enhance the implementation of psychological skills for long-term behavior change [67–68]. Future research should aim to extend the current findings by exploring other specific sports with different task characteristics and complexity. Exploring temporal changes in the use of basic and more advance psychological skills using an intervention approach while manipulating competitive anxiety to determine whether the type and function vary as time to competition moves closer would be worthwhile.

Taken together, the findings show that elite performers possess a repertoire of psychological skills that can be drawn upon during the time preceding competition. Additionally, it is also clear that some psychological skills vary within the temporal period and that elite athletes increase or decrease their range and scope of psychological skills used as competition moves closer. Utilization of these skills may influence elite performers’ attainment and maintenance of positive pre-performance psychological state compared to their national counterparts.

Even though the present study enhances our understanding of psychological preparation prior to competition, we acknowledge some possible issues in the interpretation of the findings. It would be very spurious to infer the current conceptualization of psychological skills usage through the utilization of the TOPS scale. Specifically, the fact that it only purports to measure the amount an individual utilizes a psychological skill, and does not consider whether the performer perceives he/she is actually using that skill effectively is a concern [5]. For example, an athlete’s continuous adoption of a somatically based progressive muscular relaxation strategy (e.g., dynamic stretching) may not guarantee its effectiveness in combating anxiety symptoms due to an incorrect technique chosen. Therefore, future research into psychological skills should examine not only the frequency of usage but also the perceived effectiveness of usage of different skills through the intervention approach. Scope exist for comparing the efficacy and effectiveness of one strategy against the other to facilitate performance improvements [4].

The specific nature of the population and sport under investigation limit generalization to other groups of elite and sub-elite athletes. Specifically, the current sample comprised of male and female athletes of Ghanaian nationality who participated in an interactive sport (table tennis). The characteristics of this sport may require psychological needs not found in other disciplines. These sports may require the use of different psychological skills not revealed by the current sample.

In conclusion, understanding psychological skills deployed by elite athletes can provide some foundation on which to develop and effectively implement psychological skills training programs. The current sample of athletes had no prior experience of psychological skills training or other formal forms of psychological support. Therefore, any psychological skills reported by these athletes may have been learnt intuitively through their experience of playing table tennis (or other sports) or modeled by significant others within their sporting context. The current results support previous studies that found support for mental techniques developed through natural learning experiences [30,69]. Sport psychology consultants, coaches and other analogous personnel who work with these athletes should institute formalized psychological skills training programs to build on already existing mental skills in athletes’ repertoire to develop more elaboration.
